# Understanding Viral and Immune Interplay During Vertical Transmission of HIV: Implications for Cure

**DOI:** 10.3389/fimmu.2021.757400

**Published:** 2021-10-21

**Authors:** Omayma Amin, Jenna Powers, Katherine M. Bricker, Ann Chahroudi

**Affiliations:** ^1^ Department of Pediatrics, Emory University School of Medicine, Atlanta, GA, United States; ^2^ Center for Childhood Infections and Vaccines of Children’s Healthcare of Atlanta and Emory University, Atlanta, GA, United States

**Keywords:** HIV, MTCT, cure, pediatric, shock and kill, antiretroviral, breast milk, reservoir

## Abstract

Despite the significant progress that has been made to eliminate vertical HIV infection, more than 150,000 children were infected with HIV in 2019, emphasizing the continued need for sustainable HIV treatment strategies and ideally a cure for children. Mother-to-child-transmission (MTCT) remains the most important route of pediatric HIV acquisition and, in absence of prevention measures, transmission rates range from 15% to 45% *via* three distinct routes: *in utero*, intrapartum, and in the postnatal period through breastfeeding. The exact mechanisms and biological basis of these different routes of transmission are not yet fully understood. Some infants escape infection despite significant virus exposure, while others do not, suggesting possible maternal or fetal immune protective factors including the presence of HIV-specific antibodies. Here we summarize the unique aspects of HIV MTCT including the immunopathogenesis of the different routes of transmission, and how transmission in the antenatal or postnatal periods may affect early life immune responses and HIV persistence. A more refined understanding of the complex interaction between viral, maternal, and fetal/infant factors may enhance the pursuit of strategies to achieve an HIV cure for pediatric populations.

## Introduction

Pediatric AIDS was first described in 1982, shortly after the first adult cases were reported (1). Of the estimated 37.5 million people currently living with HIV-1 as of 2020, 1.7 million of those individuals are children (2). While heterosexual transmission is the major transmission mode in adults, the majority of pediatric infections occur through mother-to-child transmission (MTCT) during pregnancy, labor and delivery, and postpartum through breastfeeding. Tremendous progress has been made in both prevention and treatment of HIV in children. However, despite the implementation of prevention of mother-to-child transmission (PMTCT) measures that decrease the risk of vertical HIV transmission to less than 5% (3, 4), approximately 150,000 children were newly infected with HIV in 2020, the majority of whom live in sub-Saharan Africa (5). As with HIV infection in adults, the primary targets for infection are activated CD4+ T cells expressing the CCR5 co-receptor. Risk of MTCT transmission is influenced by geography, maternal viral load, co-infections, delivery mode, and breast-feeding (6), among other factors. The precise mechanisms of MTCT and how its timing influences establishment of HIV infection and persistence have not been fully elucidated. In this review, we aim to summarize what is known about HIV MTCT, the immunologic and virologic factors involved in HIV transmission in the fetal and neonatal/infant periods, and provide implications for HIV cure strategies targeting perinatally infected children.

## Routes and Mechanisms of Vertical Transmission

Mother-to-child transmission (MTCT) remains the most important route of pediatric HIV acquisition and, in the absence of preventative measures, transmission rates range from 15% to 45% *via* three distinct routes: *in utero*, intrapartum, and in the postnatal period through breastfeeding. Overall, it is estimated that 20-25%, 35-50% and 25-45% of perinatal HIV transmissions occur *in utero*, intrapartum, and through breastfeeding, respectively (7–9). It is important to note that despite the prolonged exposure to HIV during fetal development, delivery, and breastfeeding, MTCT of HIV is relatively inefficient, and many children born to women with HIV do not become infected, even in absence of preventative services. Here, we will discuss the biological mechanisms of HIV MTCT and with emphasis on the factors influencing transmission. While this is still an active area of research, further elucidating these mechanisms of transmission is likely to inform cure approaches that should be considered for the almost 2 million children living with HIV.

### *In Utero* Transmission

Transmission of HIV *in utero* is the least efficient route of MTCT, accounting for an estimated absolute rate of 5-10% of MTCT for women not receiving antiretroviral therapy (ART) (7, 8). The major risk factors for *in utero* transmission are high maternal viral loads and placental inflammation (8, 10, 11). The mechanisms of *in utero* HIV transmission are incompletely understood, but some have been proposed. Early studies suggested that *in utero* transmission may occur though HIV in the amniotic fluid coming into contact with fetal mucosal surfaces (12). Although the fetal gastrointestinal tract is populated with targets for HIV infection (e.g., CD4+CCR5+ T cells) (13), more recent studies have found that even in women with detectable virus in the plasma, HIV is not detected in the amniotic fluid (14–16). Another study found that amniotic fluid has innate inhibitory activity against the replication of HIV (17). Consequently, it is more likely that *in utero* transmission occurs primarily through the placenta. The placenta is a highly effective barrier that successfully inhibits most pathogens from reaching fetal circulation through various protective mechanisms. The placenta is composed of fetal-derived trophoblast progenitor cells that differentiate into specialized cell layers. These specialized trophoblasts have broad antiviral activity in addition to acting as a physical barrier to pathogens (18). Maternal blood comes into direct contact with the placenta by 10-12 weeks of gestation when trophoblasts form the placental villi and mediate the exchange of gases, nutrients, and waste products between maternal and fetal tissue (18).

While *in utero* transmission of HIV has been documented to occur as early as 8 weeks gestation (19), the vast majority of transmissions *via* this mode occur in the third trimester (20, 21). There are two mechanisms of transplacental transmission: infection of the trophoblasts and transcytosis across the trophoblastic layer. Although trophoblasts have low to no expression of CD4 receptors, multiple studies have detected HIV in these cells (22–24). More recent evidence suggests that transmission occurs primarily through cell-associated virus, as trophoblasts appear to be naturally nonpermissive to cell-free HIV (25). In a study designed to mimic the complex cellular architecture of the placenta, transcytosis and infection of the trophoblastic layer were only accomplished by cell-associated virus (26). New evidence suggests that cell-associated HIV enters trophoblasts *via* fusion with maternal lymphocytes (27). This cell-fusion mediated spread of HIV may be less sensitive to ART than cell-free infection, causing the placenta to become a potential reservoir for the virus during pregnancy (27). It is important to note that even when HIV successfully traverses the placenta, transmission to the fetus is not guaranteed. Studies have shown that both infected and uninfected infants had maternal cells with HIV DNA in their dried cord blood, and there was no significant difference between the rate of infection and the presence of HIV-infected cells in cord blood (28, 29). These findings suggest that there are likely more factors, some of which we will discuss in later sections of this paper, governing *in utero* transmission of HIV.

### Intrapartum Transmission

The most common route of HIV MTCT occurs during labor and delivery, accounting for an absolute rate of 10-20% of infections in children born to women not receiving ART (7, 8). A major risk factor for intrapartum transmission is again maternal viral load (30–32), and while in the United States the current standard of care is delivery by Caesarian section unless women are on ART with low viral loads, most women deliver vaginally elsewhere in the world (33). Although multiple mechanisms of intrapartum transmission have been proposed, most of the evidence supports infection *via* exposure of infant mucosal surfaces to maternal secretions and blood during birth. The fetal intestines are populated with high levels CD4+CCR5+ T cells (13), and evidence suggests that higher viral loads in the birth canal correlate with increased transmission rates (10). Additionally, factors that would increase contact of fetal mucosal surfaces with maternal viral secretions also increase transmission rates. For example, genital ulcers have been shown to cause an increase in intrapartum transmission rates (34). This association remained significant even after adjusting for plasma HIV viral load. Several possible mechanisms are thought to explain this association including increased recruitment of HIV-infected CD4+ T cells to the mucosal surface of the genital lesions resulting in increased viral load in the genital tract (35, 36). Finally, the protective effect of elective Caesarian section against MTCT of HIV is attributed to the reduction of fetal contact with the birth canal (37–39).

Another proposed mechanism of intrapartum transmission is maternal-fetal microtransfusion (also called placental microtransfusion) causing fetal exposure to maternal blood containing both cell-free HIV RNA and cell-associated HIV DNA. Although the cause is unknown, microtransfusions occur at or near time of delivery when there is a disruption of the placental barrier and are thought to increase when uterine contractions intensify causing membranes to rupture. One study done using placental alkaline phosphatase (PLAP) as a marker for microtransfusions found that in women who had vaginal deliveries, high levels of PLAP were associated with higher intrapartum transmission risk (40). However, there was no significant association between PLAP (microtransfusion) levels and perinatal transmission as a whole. As described above, further studies designed to detect maternal cells in dried fetal cord blood as a proxy for microtransfusion also found no correlation (28, 29). Maternal-fetal microtransfusions are still relatively understudied, however.

### Postpartum Transmission

Breastfeeding contributes to a considerable proportion of pediatric HIV infections accounting for an estimated absolute rate of 5-15% of MTCT for women not receiving ART (8). Premastication, where an adult chews foods before feeding it to the child, is also a risk factor for MTCT and this risk is associated with predisposing oral conditions that lead to the presence of blood in the mouth (41). It was recognized early on that breastfeeding was a potential mechanism for HIV transmission; however, the World Health Organization recommends that women in sub-Saharan Africa (with high HIV prevalence) breastfeed their children due to lack of consistent access to a safe water supply (42). Other factors such as the cost of formula and the stigma associated with not breastfeeding play into this decision (7). In order for MTCT to occur during breast feeding, the virus must first pass through the mammary epithelium, remain infectious within breast milk, traverse the infant mucosal barriers and establish infection. Studies in *ex vivo* organ tissue model systems and *in vivo* animal models have provided evidence that transmission can occur *via* the oral route (43, 44). Oral inoculations of simian immunodeficiency virus (SIV) have been shown to cause infection in infant rhesus macaques, providing proof of concept, although often these experimental inoculums contained higher doses of virus than would be seen in normal levels in breast milk (45–47). Cord blood and neonatal blood contain low levels of CD4+ T cells expressing CCR5, but, as described above, CD4+CCR5+ T cells populating the fetal intestines are abundant (13), a finding that has also been described in infant rhesus macaques (48–50). The mammary epithelium, like the placenta, however, successfully inhibits many pathogens from entering the breast milk through various protective mechanisms. Additionally, there is evidence that breast milk and saliva have innate inhibitory activity that restricts HIV transmission (51–54). The protective mechanisms of the maternal and fetal immune responses that limit breast milk transmission will be discussed in more detail below.

One major risk factor for MTCT during breastfeeding is viral load in the breast milk (10), and both cell-free and cell-associated HIV are thought to contribute to transmission. HIV RNA levels in breast milk correlate with plasma levels but are generally 100-fold lower (8, 55). Conditions that increase breast milk viral shedding are associated with higher transmission rates. Nonexclusive breastfeeding and infrequent emptying of the breast can lead to breast inflammation secondary to milk stasis, which in turn increases the viral load and has a strong association with increased MTCT (56–61). However, conflicting research found that nonexclusive breast feeding and mastitis do not significantly increase viral loads in breast milk (56). Still, HIV transmission was increased in breastfed infants who also received solid foods when compared to exclusively breastfed children (58). This mixed feeding is thought to cause disruption of the infant’s gut mucosal lining secondary to the introduction of non-breast-milk foods and early introduction of pathogens or foreign antigens, leading to immune activation and increased susceptibility to infection (62–64). Microbiome differences between mixed fed and exclusively breastfed infants may also impact transmission (65). Multiple studies suggested that cell-associated viruses also have a role in transmission during breastfeeding (66, 67). Understanding these biological mechanisms of breast milk viral spread is an important first step in characterizing the establishment of HIV reservoirs during this period. Further, it should be recognized that breastfeeding transmission can occur outside of the neonatal period and infants may be diagnosed after more time has elapsed since infection than occurs with *in utero* and intrapartum transmission. This later diagnosis, coupled with rapid immune system changes in early life, likely leads to a unique immunovirologic environment defining HIV persistence in children infected through the breastfeeding route.

## Factors Influencing Vertical Transmission

**Figure 1:**
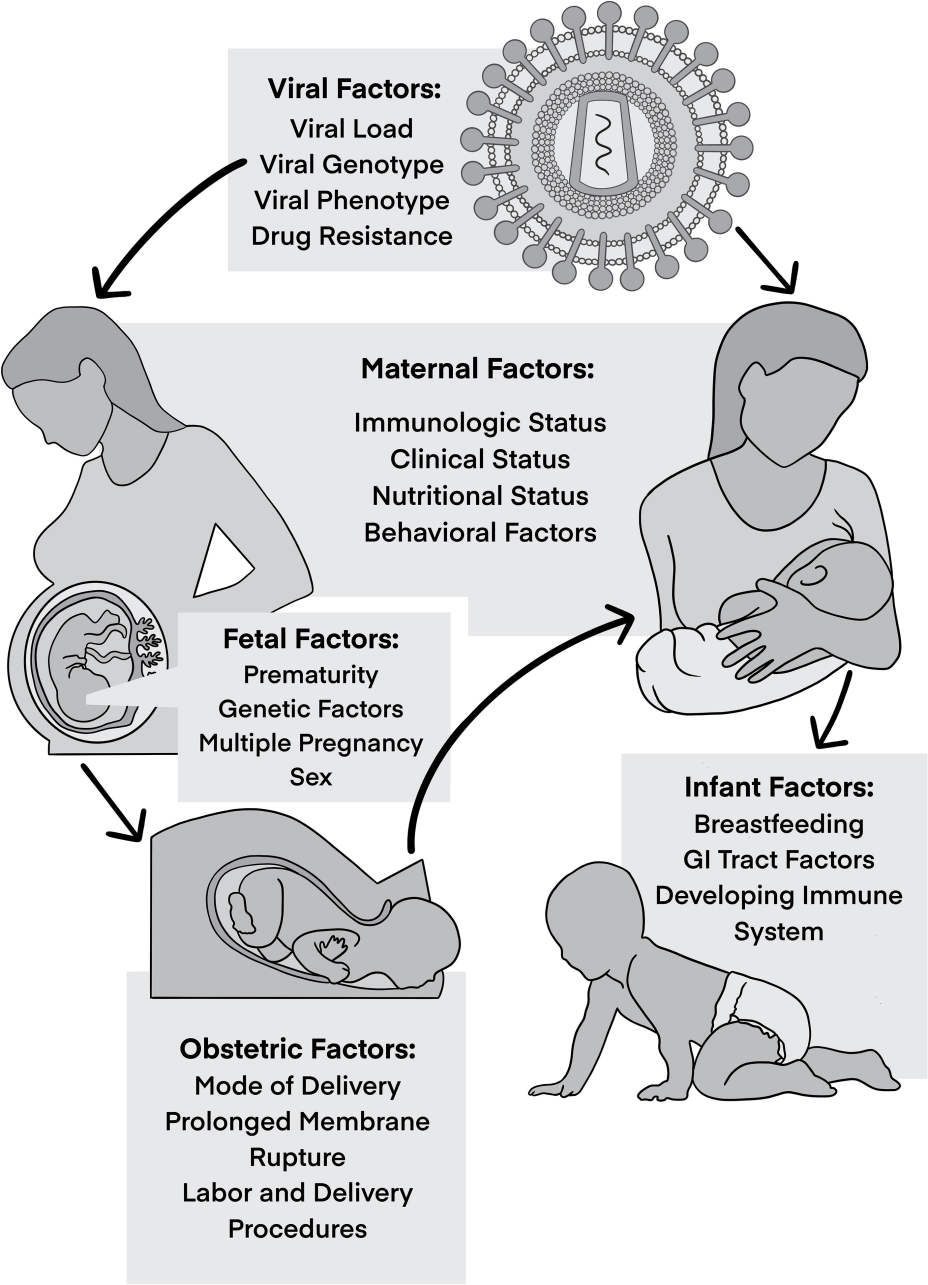
Factors influencing vertical transmission.

MTCT is multifactorial, with different viral, maternal, and fetal/infant factors at play that influence the risk of HIV transmission (**Figure 1**). Knowledge of these risk factors and understanding of their roles in MTCT have been crucial to develop preventative measures and here we postulate that their consideration should also inform the investigation into curative approaches.

### Viral Factors Impacting MTCT

#### Viral Burden

Maternal viral load has been shown to be the strongest predictor of perinatal HIV transmission. Many studies have demonstrated increased risk of transmission with high levels of maternal viremia and high p24 antigenemia (68–71). Acute infection during pregnancy is associated with increased risk of perinatal transmission and is likely related to the high viral loads in plasma and the genital tract (72). Consequently, maternal viral suppression *via* use of ART during pregnancy has been proven to lower the risk of perinatal transmission. This was first supported by the 076 trial which showed that administration of zidovudine during pregnancy, delivery, and to the newborn for the first 6 months of life reduced transmission by nearly 70% in women with HIV who did not breast-feed (73). Subsequent studies have indicated that transmission is reduced even further with triple-drug regimens (74). Several studies have tried to establish a threshold of maternal viral load below which transmission does not occur (75); however, it has been demonstrated that transmission can occur in some cases where women have low levels of HIV RNA in the blood, indicating the presence of other factors influencing the transmission process. In one study that included 320 women from 18 different centers in France showed that perinatal transmission occurred in 12% of the women at less than 1000 copies/ml compared to 29% in women with more than 10,000 copies/ml (76). In contrast, in Bangkok 281 pregnant women were followed and no transmission occurred in those with virus load less than 2,000 copies/ml (77).

Several behavioral practices have been associated with increased risk of maternal to infant HIV transmission due to resultant increases in viral load. These behavioral factors include illicit drug use (IDU), cigarette smoking, and alcohol use. IDU is associated with increased risk of MTCT (78–81). Alcohol and drug use like heroin, amphetamine and cocaine, may be associated with failure to control viral load in the presence of ART, hence increased transmission rate (82–85). Additionally, transmission may be increased through drug interactions with ART and placental injury (86). Some have demonstrated an association between IDU and lack of adherence to ART which can lead to increased viral load (83, 85–87).

In addition to plasma viral load, viral levels in cervicovaginal fluid and breast milk are also thought to influence transmission risk in the intrapartum and postnatal periods (56, 88–90). As referenced above, levels of HIV DNA in breast milk cells (ie, CD4+ T cells) are positively correlated with postnatal transmission, and this intracellular HIV DNA is not significantly reduced by maternal ART. Risk of transmission is higher during early lactation due to the increased cellular content of colostrum (91). Both maternal ART and extended infant prophylaxis (at least with a single agent) during breastfeeding significantly reduces but does not eliminate MTCT, an observation that likely reflects virus persistence in CD4+ T cells (92–97).

#### Viral Genotype and Phenotype

Several studies of viral variants in mothers and infants attempted to characterize HIV quasispecies associated with MTCT and have yielded conflicting results. Major, minor, and multiple variant transmission events have been described (98–100). Most evidence suggests that a single or restricted subset of maternal viral variants establish infection in the infant suggesting selective pressure during the transmission process. However, the basis and the factors controlling MTCT bottleneck are not yet fully understood. Different properties of virus populations have been reported in studies analyzing vertical transmission stratified by timing, with transmission during the intrapartum period found to be primarily associated with the transmission of minor maternal variants while *in utero* transmission was more likely to occur with single or multiple HIV variants (100–104). Maternal antiretroviral drug resistance is associated with transmission during breastfeeding, but not during the *in utero*/intrapartum periods (105).

Phylogenetic analyses of HIV *env* in infected infants showed that they have a more homogenous virus population when compared to their mothers (106, 107). Studies have also shown that macrophage-tropic and non-syncytium-inducing (NSI) or CCR5-utilizing HIV viral strains are selectively transmitted (108). Another characteristic found in transmitted viruses was shorter variable loops and fewer putative N-linked glycosylation (PNG) sites encoded in *env* (70, 109–111). Different HIV subtypes may also have distinct MTCT rates. Work conducted in Tanzania showed that subtype C is preferentially transmitted from mother to child compared to subtype A and D (100). In another study from Kenya, the MTCT rate appeared to be higher among mothers infected with subtype D compared with subtype A (112). However, no such differences have been observed in other cohorts (100, 112–115).

### Host Factors Impacting MTCT

#### Genetic Factors

Genetic polymorphisms in the coding and regulatory regions of HIV receptors and their ligands influence the risk of HIV acquisition. Infants with a single nucleotide polymorphism (SNP) in the CD4 gene at position C868T that may modify the tertiary structure of CD4 were more likely to acquire HIV compared to infants with wild type CD4 (116). In the setting of MTCT, most of the transmitted viruses use CCR5 as a coreceptor (117). As has been shown for horizontal infection in adults, the presence of a 32-bp deletion in the coding region of the *CCR5* gene (*CCR5*-Δ32) in the homozygous state in infants results in non-functional coreceptors and confers protection from vertical infection (118, 119). Heterozygosity also exerts a protective effect when carried by mothers due to lower maternal viral burden (107). Conversely, polymorphisms in the CCR5 promoter region at positions 59029 and 59353T increase the expression of CCR5 leading to increased risk of MTCT when carried by infants (120–123).

Increased risk of vertical transmission was also seen with genetic polymorphisms resulting in decreased expression of the natural ligands for HIV coreceptors (CCL3, CCL4, and CCL5) (124).

Genetic variations affecting innate immunity may also influence MTCT. Defensins are antimicrobial peptides that are expressed by epithelial cells, known for innate mucosal defense and antiviral activities. Defensins inhibit HIV infection *via* different mechanisms including direct binding to virions as well as disturbing intracellular signaling by modulation of host cell surface receptors. Three SNPs in the 5′ untranslated region of β-defensin-1 (*DEFB1*) gene were reported to modulate risk of MTCT: −52G/A, -20(G/A) and −44C/G (125–127). Braida et al. described an association between -44(C/G) and HIV infection in Italian pediatric population (125). Another study found a lower copy number of *DEFB104* among HIV-infected children when compared to HIV-exposed children and healthy controls, suggesting that *DEFB104* may have a potential protective role against vertical transmission (128).

Multiple studies have investigated the influence of HLA concordance between mother and infant on vertical transmission and as well as risk of disease progression in infected infants (129–131). Specific maternal HLA polymorphisms, including B4901, B5301, A2/6802 and B18, have also been associated with decreased risk of MTCT (132–134). HLA-G, a non-classical class I MHC gene highly expressed in placental trophoblasts, has several SNPs found to be associated with decreased risk of vertical transmission (135, 136). Certain class II MHC alleles have also been reported to influence MTCT among certain ethnicities such as DQB1*0604, *DR3*, *DR13*, DRB1*1501 (137–139).

Gender specific differences in MTCT have been reported in several cohorts (140, 141). Female infants are reported to have two- to three-fold increased risk of infection at birth compared to male infants (7, 140, 141). This increased susceptibility has been linked to subversion of innate immunity, with female fetuses acquiring maternal variants resistant to type I interferons (142). Sex-specific differences in *in utero* infection have also been attributed to the fact that *in utero* mortality rates of HIV-infected male infants are disproportionately higher and thus more HIV-infected female infants are liveborn. It is also proposed that a minor histocompatibility reaction between infant male Y chromosome-derived antigens and maternal lymphocytes reduce the risk of MTCT to boys (140).

#### Maternal Co-Infections

Several coexisting maternal infections have been found to be associated with increased MTCT of HIV. Multiple studies demonstrated an increased risk of vertical transmission in the setting of chorioamnionitis (143–145), primarily related to disruption of the placental barrier and entrance of HIV-infected cells into the amniotic fluid (143, 144, 146). Chorioamnionitis complications including preterm labor and premature rupture of membranes can also lead to increased risk of MTCT related to immaturity of the skin and mucosal membranes as well as the premature fetal immune system. In a multicenter prospective cohort, clinical and biologic factors that contributed to MTCT were studied. Histologic chorioamnionitis was found to be a major risk factor for transmitting HIV (147).

Adachi and colleagues evaluated the effect of sexually transmitted infections (STIs) on risk of MTCT in a large cohort of HIV-infected pregnant women and found increased rates of HIV transmission in the presence of a another sexually transmitted disease (148). In general, inflammation of the maternal genital tract mucosa has been shown to increase rate of vertical transmission independent of maternal plasma HIV load (59). Infections resulting in genital ulcer disease such as HSV-2 are also associated with increased genital shedding of HIV (35, 149, 150). Conflicting data exist regarding maternal syphilis infection and MTCT of HIV. A study in Zimbabwe showed that active maternal syphilis at the time of delivery was not associated with intra-partum MTCT risk while several other groups have demonstrated an increased risk of vertical HIV acquisition in the setting of maternal syphilis (146, 151). HIV and Hepatitis B virus (HBV) co-infection are associated with increased HBV, but not HIV transmission to the infant. Maternal Hepatitis C virus (HCV) co-infection has been linked to higher rates of vertical HIV transmission (152–160).

Unprotected sexual intercourse during pregnancy itself is thought to be associated with increased risk of MTCT (161, 162). Bulterys et al. conducted a prospective cohort study in Rwanda that showed that unprotected sexual intercourse with multiple partners before and during pregnancy in a population with high HIV-1 seroprevalence may increase risk of transmission from infected mother to infants (161). Another study by Burns and colleagues also found higher frequency of intercourse during pregnancy among women transmitting HIV to their infants (163). Potential mechanisms include increased HIV strain diversity (i.e., superinfection) (164, 165) and vaginal or cervical inflammation due to microabrasions or STIs that result in chorioamnionitis and/or increased viral shedding in genital fluids (164).

Tuberculosis (TB) is one of the most important causes of mortality and morbidity in HIV infection, especially in women residing in TB endemic areas. Active TB infection increases HIV viral load which is a known risk factor for perinatal transmission (166–168). Gupta et al., found a 2.5-fold increase in the odds of MTCT of HIV in pregnant women with TB/HIV coinfection, after adjusting for maternal and infant factors (169). Similarly, malaria and HIV coinfection is associated with an increased risk of adverse outcomes in pregnant women (170) as well as increased HIV viral load (171). As such, a few studies have suggested an increased risk of perinatal transmission with maternal malaria (172–174), yet others failed to demonstrate this relationship (144, 175, 176). Finally, other common viral infections, such as Cytomegalovirus (CMV), may play a critical role in influencing MTCT. One described mechanism has been through enhancement of placental susceptibility to HIV infection (177).

#### Maternal Immunological Factors

There are multiple maternal immunological factors associated with increased risk of perinatal transmission, including low CD4+ T cell count, CD4+ T cell percentage, and CD4/CD8 ratio (178). Shivakoti et al. showed that high maternal soluble CD14 concentration during the peripartum period was associated with increased risk of MTCT, independent of maternal viral load, CD4+ T cell count and ART exposure (179), implicating immune activation in transmission.

Adaptive immune responses play an important role in transmission from mother to infant. MTCT is a unique setting where HIV-1 acquisition occurs in the presence of naturally elicited HIV-specific antibodies that are passively transferred prior to birth. Several studies have examined the impact of maternal antibodies on MTCT; however, there have been conflicting results and their role in protecting infants against HIV-1 transmission remains unclear. These contradicting results may be explained by small cohort sizes, lack of control for the other known risk factors for HIV transmission, timing, and methods used in infant diagnosis, and potential clade-specific differences in virus–antibody interactions. Initial studies demonstrated that higher levels of maternal HIV-1 envelope (Env)-specific IgG antibody responses were associated with reduced transmission risk (180, 181).This association was not observed in subsequent studies and research focus was shifted to examine the effect of neutralizing antibodies (nAbs) and their role in modulating the risk of transmission (182, 183). Work conducted in nonhuman primates showed that passive immunization of infants with a cocktail of HIV-1-neutralizing antibodies provided partial protection against oral simian-human immunodeficiency virus transmission (184). Subsequent human studies showed that high levels of maternal nAbs were correlated with reduced MTCT rates while others failed to confirm this association (185–188). However, founder viruses in infants are generally more resistant to neutralization by maternal antibodies (189, 190), suggesting that the transmitted variants are able to escape nAbs and supportive of the idea that maternal antibodies may confer partial protection.

In a large cohort of non-breastfeeding HIV-1 infected women enrolled in the pre-ART era Women and Infant Transmission Study (WITS), Permar and her group found that maternal V3-specific IgG binding responses and CD4 binding site-blocking responses correlated and were independently predictive of reduced MTCT risk (191). They also found that both binding and neutralizing responses targeting the C-terminal region of HIV envelope (Env) were associated with decreased risk of transmission (191). Antibody effector functions beyond neutralization have also been examined for their contribution to MTCT. Maternal antibody-dependent cellular cytotoxicity (ADCC) is thought to have a role in protection against HIV-1 transmission. In one such investigation, plasma and breast milk obtained soon after delivery from 9 transmitting and 10 non-transmitting women in Kenya demonstrated that breast milk Env-specific IgG responses with ADCC activity were associated with decreased MTCT risk (192). However, Pollara et al. found no association of ADCC-mediating responses and MTCT risk in HIV clade C breastfeeding women in Malawi (193). In addition to antibodies and antibody effector functions, Lohman-Payne et al. also reported breast milk HIV gag-specific IFNg responses to be associated with protection from MTCT *via* breastfeeding in a Kenyan cohort (194).

#### Infant Immunological Factors

Most infants born to HIV-1 infected mothers do not become infected despite having an immature immune system and repeated exposures to HIV-1 throughout the peripartum and breastfeeding periods. To survive to term, the developing fetus must avoid generating an inflammatory response to the many foreign maternal antigens to which it is exposed during development resulting in a predominantly tolerogenic immune system in the fetus and newborn (195). The tolerogenic environment is facilitated by high levels of anti-inflammatory cytokines such as TGFb and IL-10 (196), which likely subvert immune activation and establishment of infection upon HIV exposure. TGFb also directs naïve CD4+ T cell to differentiate to Tregs resulting in a larger Treg pool in infants, representing 15% of fetal blood T cells compared to 5% of adult blood T cells. These Tregs promote immune tolerance and are long-lived (196, 197). Much of these regulatory cells reside in intestinal tissue and are critical for mucosal immune homeostasis. In addition to higher levels of Tregs, the CD4+ effector cells of infants are predominantly of the Th17 and Th2 phenotype (rather than Th1 as in adults) (198, 199). Th17 cells are critical to maintaining the integrity of the intestinal mucosal barrier and may thus restrict dissemination of infection. Conversely, Tugizov and colleagues have reported lower levels of innate proteins that restrict infection, such as defensins, in infant compared to adult oral epithelia (43).

In the mother-child transmission pair, the infant may be disadvantaged in that the transmitted virus in has already adapted to evade a genetically similar immune system. Anti-HIV-1 antibodies and T cells transferred to the child either *in utero* or through breastmilk have pre-adapted to the transmitted virus and may be ineffective. Additionally, because of shared HLA alleles the transmitted virus may be preadapted to escape CD8+ T cells targeting HIV epitopes restricted by HLA alleles inherited from the mother further complicating pediatric HIV-1 infection (200, 201). HIV-specific cellular immune responses are detected in exposed uninfected infants, but their role in influencing virus acquisition is uncertain (202, 203).

## Implications for Cure Approaches

The World Health Organization recommendations state that all infants and children under two years of age with confirmed HIV-1 begin ART immediately at the time of diagnosis irrespective of CD4+ T cell counts (204). Despite this recommendation, HIV-1-infected children are one third less likely to receive ART than infected adults (205). Reasons for this include fewer drugs available for use by children, higher treatment cost, and dependence on a caregiver to provide ART (206). These factors can complicate the design and implementation of cure studies in children.

A major barrier to HIV cure is the establishment of a reservoir of HIV-infected cells that persists despite suppressive ART and can give rise to rebound viremia if ART is interrupted. In adults, it is well documented that viral reservoirs are established during early stages of HIV infection (207) and this finding is supported by the SIV/macaque model in which rapid seeding of the viral reservoir within 3 days of infection has been described (208). Furthermore, rebound of viremia is almost always seen after ART discontinuation in both horizontally infected adults and perinatally-infected children (209–211). However, little is known about the exact timing of reservoir seeding in different MTCT settings and whether there are differences in reservoir establishment in relation to time of infection and mode of transmission. Increased understanding of these factors may aid in the development of approaches to eradicate viral reservoirs and/or induce viral remission (sustained viral suppression in absence of ART) in children.

The uniqueness of the in-utero transmission window allows for rapid detection and treatment as children born to women with HIV can be screened at birth. Such early detection is not generally feasible in adult transmissions. Infants diagnosed at birth *via* rapid point-of-care testing can begin ART within the first few hours of life (212). Infections arising from intra- or post-partum transmission will not be detected at the time of birth, so follow-up care and repeated testing are required to monitor these transmission modes. In these instances, early ART initiation is not as practical in resource limited settings, although current United States guidelines recommend triple drug prophylaxis (given at therapeutic dosing) for neonates with high risk of acquiring HIV infection.

Contemporary studies of HIV reservoirs in perinatally infected infants and children have focused on *in utero* transmission, following the description of the ‘Mississippi child’. This child received ART between 30 hours and 18 months of age, and then remained persistently aviremic for 27 months after discontinuation of ART before rebound (209, 213). *In utero* infection was documented, but the child did not develop HIV-specific antibody or T cell responses, thought due to the very early initiation of ART. Despite the eventual rebound, this partial remission led to several clinical trials of very early ART (typically considered to be within 48 hours) to limit the size of the HIV reservoir and investigate the possibility of achieving post-treatment control after ART interruption. Results from the Early Infant Treatment (EIT) study in Botswana have demonstrated that very early ART leads to an exceptionally small reservoir of intact proviral HIV DNA as well as an improved innate and adaptive antiviral immune response compared to delayed ART (214). As of this writing, results from the ART interruption phase of the very early ART trials, including EIT and IMPAACT P1115 (215), have not been published. Kuhn and colleagues describe challenges in meeting predetermined virologic and immunologic criteria for ART interruption in the LEOPARD trial of very early/early ART conducted in South Africa (216).

The South African Children with HIV Early antiRetroviral therapy (CHER) trial (217), that included children with *in utero* and intrapartum HIV infection, has led to a number of key findings regarding persistent HIV reservoirs. One report demonstrated rare intact proviral sequences in children after 6-9 years of ART initiated after 2 months of age but within the first year of life using near full-length proviral amplification and sequencing (218). This group further identified clones of infected cells shortly after birth that could also be detected after almost a decade on ART, suggesting that clonal expansion of reservoir cells maintains HIV persistence in children as it does for adults (219). Two additional case reports of long-term HIV remission have emerged, one from the CHER trial, with unknown timing of infection (*in utero vs* intrapartum) (220) and the other from a French cohort, with presumed intrapartum transmission (221), both of whom started ART at 2-3 months of life.

There have been relatively fewer studies of reservoir characteristics in children infected postpartum through breastfeeding. However, presently over half of new infections occur postnatally through breast milk (9). As this route has become predominant in perinatal HIV infection, a nonhuman primate model has been established to better understand virologic and immunologic features of lentivirus infection following postpartum transmission, more precisely define anatomic sites of virus persistence, and test strategies to promote reservoir eradication or remission (46, 47, 222–224). These and other ongoing studies may inform the design of future cure-directed clinical trials in children with HIV infection acquired through breast milk.

Regardless of the mode of transmission earlier ART is generally associated with a smaller reservoir during viremia suppression (210, 214, 225–237), although starting ART within 14 days of life may not lead to significantly greater HIV DNA persistence compared to within 48 hours of life (238). ART initiation within 8 days has also been associated with a faster HIV DNA decay compared to ART started at 5 months (239). Female sex and maternal acute HIV infection during pregnancy have been associated with higher levels of persistent HIV DNA (240).

While protocol-specified ART interruption has not occurred in recent clinical trials, studies of intermittent viremia *vs*. sustained viral suppression can be informative to identify biomarkers that may be predictive of viral rebound dynamics. These findings are by nature complicated by behavioral factors, including adherence to ART regimens. Millar and colleagues assessed factors associated with intermittent viremia in *in utero* infected infants started on early ART (in the first 3 weeks of life) (240). A smaller reservoir size as measured by total HIV DNA in PBMCs was not correlated with maintained viral suppression whereas a longer time to initial viral suppression was, implicating immunologic contributions to aviremia, although this finding may have been influenced by ART nonadherence. In children in the EIT study who started on ART at < 7 days of life, sustained HIV RNA suppression was associated with negative HIV serostatus and negative qualitative HIV DNA PCR at ~20 months (225). HIV seronegativity has also been described in other studies of early or very early ART and has been proposed as an estimate for reservoir size (227, 228, 241–244).

**Figure 2:**
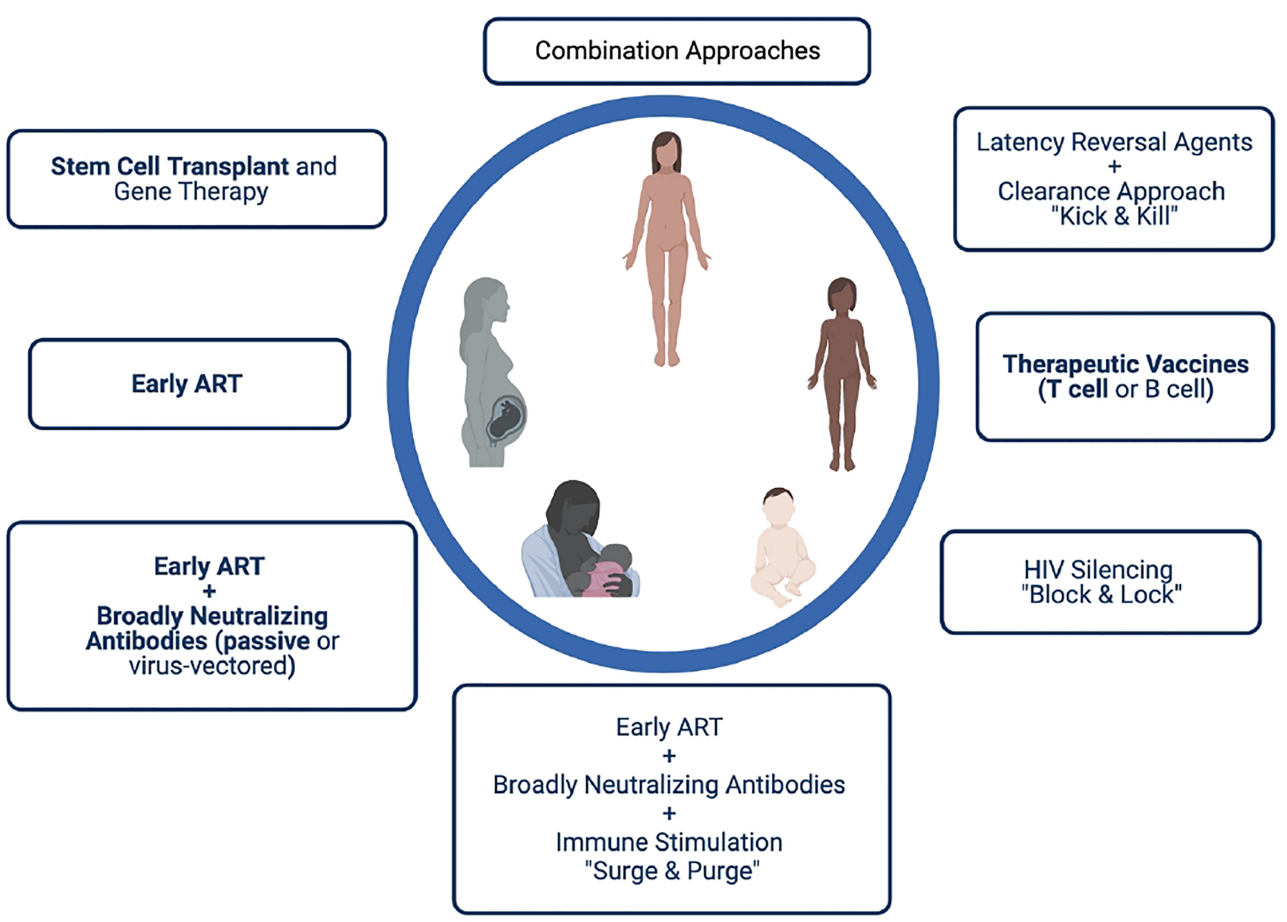
Possible HIV cure approaches for perinatally infected infants, children and adolescents. Strategies in bold have been or are being studied in pediatric clinical trials.

Identification of cure strategies that target latently infected cells, allow immune recognition and clearance of the reservoir, and/or promote viral remission are currently considered extremely high priority for the field. Unlike in adults, few cure strategies have been tested in children to date, with the exception of very early ART that it is now understood is insufficient to lead to cure in the majority of individuals. However, there are several approaches that may provide benefit in the setting of perinatal HIV transmission, and their study should be carefully considered for certain populations (**Figure 2**). The design of such studies should take into consideration not only the timing of ART initiation, but also the duration of ART (and therefore age of the trial participant), as well as the known or presumed mode of HIV transmission (*in utero*, intrapartum, or postpartum). For example, a neonate infected *in utero* and initiating very early ART is likely to be vastly different both virologically and immunologically from an adolescent who acquired HIV through breastfeeding with delayed ART initiation and periods of unsuppressed viremia over years. In the former case, adding the cure directed therapy at the time of early ART initiation may lead to an extremely small reservoir size (or even prevent reservoir establishment in the best-case scenario). This cure-directed therapy might include one or multiple broadly neutralizing antibodies delivered by passive (as is being tested in IMPAACT 2008 (215) or active immunization along with ART. A novel proposed strategy, termed “surge and purge,” combines very early ART, passive antibody administration, and immune stimulation to destabilize reservoir establishment (245). Infants and children with very small reservoirs may also be good candidates for approaches designed to silence HIV expression (“block and lock”). For the school aged or adolescent child with a larger reservoir size, the “kick and kill” approach may be more effective, with a latency reversal agent used to reactivate virus expression followed by immune-based clearance of infected cells. Studies in nonhuman primates support the notion that pre-existing reservoir size as well as age-related factors may influence susceptibility to latency reversal (246, 247). Therapeutic HIV vaccines designed to boost antiviral T cell responses may have benefit in kick and kick strategies targeting established infection (223, 248) and possibly in limiting reservoir size early in infection, although in both settings the impact may depend on the child’s age at vaccination, the timing of ART, and the degree of immune exhaustion present. Results from the HVRRICANE trial are forthcoming, in which a prime-boost vaccine strategy will be tested in combination with a TLR4 adjuvant (hypothesized to reverse latency) in HIV-1-infected children with history of early ART or previous immunization with one of the vaccine components (215).

In summary, HIV MTCT is promoted by distinct immunopathogenesis for the three different routes of transmission that may also affect HIV persistence. Consideration of the complex interaction between viral, maternal, and fetal/infant factors may enhance the pursuit of strategies to achieve an HIV cure for pediatric populations. Future preclinical and clinical trials from the neonatal period through adolescence will further elucidate how MTCT influences the potential for HIV cure.

## Author Contributions

OA is the first author, the rest of the coauthors AC, KB, JP contributed to the manuscript. All authors reviewed the final manuscript. All authors contributed to the article and approved the submitted version.

## Funding

AC would like to acknowledge funding support from the NIH (R01 AI133706, P01 AI131276, R37 AI157862, and UM1 AI164566).

## Conflict of Interest

The authors declare that the research was conducted in the absence of any commercial or financial relationships that could be construed as a potential conflict of interest.

## Publisher’s Note

All claims expressed in this article are solely those of the authors and do not necessarily represent those of their affiliated organizations, or those of the publisher, the editors and the reviewers. Any product that may be evaluated in this article, or claim that may be made by its manufacturer, is not guaranteed or endorsed by the publisher.
